# Removal efficiency of a fast setting pozzalan-based bioactive cement: a micro CT study

**DOI:** 10.1186/s12903-024-04546-6

**Published:** 2024-07-11

**Authors:** Feyza Çetinkaya, Ahter Şanal Çıkman, Ali Keleş, Banu Arıcıoğlu

**Affiliations:** 1 Department Of Endodontics, Recep Tayyip Erdoğan University Faculty Of Dentistry, Rize, Turkey; 2 Department Of Endodontics, Abant İzzet Baysal University Faculty Of Dentistry, Bolu, Turkey; 3 Department Of Endodontics, İstanbul Medeniyet University Faculty Of Dentistry, İstanbul, Turkey; 4 Bandırma Oral and Dental Health Center, Balıkesir, Turkey

**Keywords:** MTA, Regeneration, Ultrasonic, Solutions

## Abstract

**Aim:**

The aim of this study was to evaluate the removal efficiency of PRMTA and ECMPremixed applied to the coronal third according to the RET by UI and to examine the effect of different solutions on material removal.

**Materials and methods:**

40 permanent upper central teeth were used to simulate immature teeth. The samples were irrigated with 1.5% NaOCl and calcium hydroxide was placed. Samples were incubated in PBS. Then irrigation was done with 17% EDTA, the samples were randomly divided into 2 groups (*n* = 20):Group 1: PRMTA, Group 2: ECM Premixed. The materials were placed in the samples. Then the samples were scanned with micro-CT. Materials were removed by UI. Micro-CT scan of the samples was performed. Each material group was divided into 2 subgroups (*n* = 10): Group1 was MTAD, group2 was irrigated with 10% CA; then micro-CT was performed. Obtained images were positioned in DataViewer and analyzed with CTAn. The obtained data were statistically analyzed in IBM SPSS 25. The significance level was determined as 5%.

**Results:**

There was no significant difference between the initial volumes of the materials (*p* > 0.05). The amount of the remaining material after UI was significantly higher in the PRMTA (0.7471%) group compared to the ECM Premixed (0.0093%). There was no significant difference in terms of remaining material after irrigation with MTAD and CA in both groups (*p* > 0.05).

**Conclusion:**

A great deal of the materials were removed by UI under the operation microscope. ECM Premixed was removed more effectively compared to the PRMTA. And, acidic solutions did not provide any additional benefit in material removal.

## Introduction

Nowadays, regenerative approaches are increasing in endodontics area, and tooth survival with a conservative approach is becoming increasingly important, especially in necrotic immature teeth. Necrotic immature teeth have short roots with thin dentin walls, which affects their survival. The ability to continue root development by replacing damaged tissues has made regenerative endodontic treatment (RET) widespread in these teeth [1].

Calcium silicate-based materials (CSBMs) are innovative materials developed in the field of endodontics, known for their ability to promote tissue repair and stimulate mineralization, and have high sealing ability. Considering these features CSBMs are commonly used as coronal barrier materials in RET [2]. ProRoot MTA (PRMTA) is the most preferred material for use as a coronal barrier due to its sealing, biocompatibility, marginal adaptation and antibacterial properties. However, the search for new materials continues due to its disadvantages such as long curing time, discoloration and difficulty in manipulation [3]. Endocem MTA Premixed (ECM Premixed), a new generation pozzolan-based CSBM, is a paste-type ready-to-use material with a fast curing time and less coloration potential than PRMTA [4]. In addition, the material has biological and osteogenic potential comparable to PRMTA [5]. It was also reported that ECM Premixed exhibited similar push-out bond strength [6] and clinical success rates to PRMTA [7]. These studies in the literature suggest that ECM Premixed is comparable to PRMTA in many respects and is suitable for clinical use.

Despite the high success rate of RET, the persistence of symptoms (pain, percussion, fistula, mobility, swelling) after treatment is considered a definite sign of failure [8]. In case of failure, complete removal of the coronal barrier material and orthograde retreatment as a conservative approach instead of surgical endodontic applications is an important step in increasing the survival rate of the tooth. Residues of the removed material may remain on the dentin wall, and adversely affect the bonding of subsequent canal filling material. Therefore, in addition to providing effective sealing, it is of great clinical importance whether it is possible to completely remove the coronal barrier materials in case of failure in RETs [8].

Despite the well-documented benefits of CSBMs, their removal in retreatment cases remains complex and can weaken dental structures, impacting prognosis [9]. The superiority of ultrasonic systems over other systems, such as rotary systems and hand files, was proven for the removal of CSBMs [10]. Additionally, some studies have shown that acidic solutions, such as 10% citric acid (CA) and Biopure MTAD (MTAD) (4% CA and tetracycline), affect the surface properties of these materials [11, 12]. Due to these effects, it is suggested that acidic solutions may enhance the removal of CSBMs. But there is still lack of literature on the evaluation of the removal efficiency of CSBMs, especially with the use of advanced imaging techniques such as micro-CT. Therefore, it was aimed to evaluate the removal efficiency of PRMTA and ECM Premixed applied as coronal barrier materials and the effect of different solutions on removal by micro-CT. The first null hypothesis of the study was that there was no difference in removal efficiency between the ECM Premixed and PRMTA. The second null hypothesis of the study was that MTAD and 10% CA irrigation would not affect removal efficiency.

This study is expected to guide clinical practice for non-surgical endodontic retreatment of failed RETs by determining the effectiveness of ultrasonic and acidic solutions in removing 2 different CSBMs used as coronal barrier.

## Material and method

This study was approved by the Medical Ethics Committee of Recep Tayyip Erdoğan University (2022/151).

The manuscript of this laboratory study was written according to the Preferred Reporting Items for Laboratory Studies in Endodontology (PRILE) 2021 guidelines.

Power analysis was performed to test the reliability of the statistical analysis of the findings. G Power 3.0.10 (University Kiel, Germany) program was used to calculate the effect size. The effect size was calculated based on the remaining volume data of CEBECI et al. [13]. and it was determined that an effect size of 0.813 d cohen [14] was sufficient for significance. When there were two different calcium silicate based materials and 2 different irrigation solution groups, the minimum number of samples was determined as 8 for each group, a total of 32, with a type 1 error of 0.05 and 99% power.

**Fig. 1:**
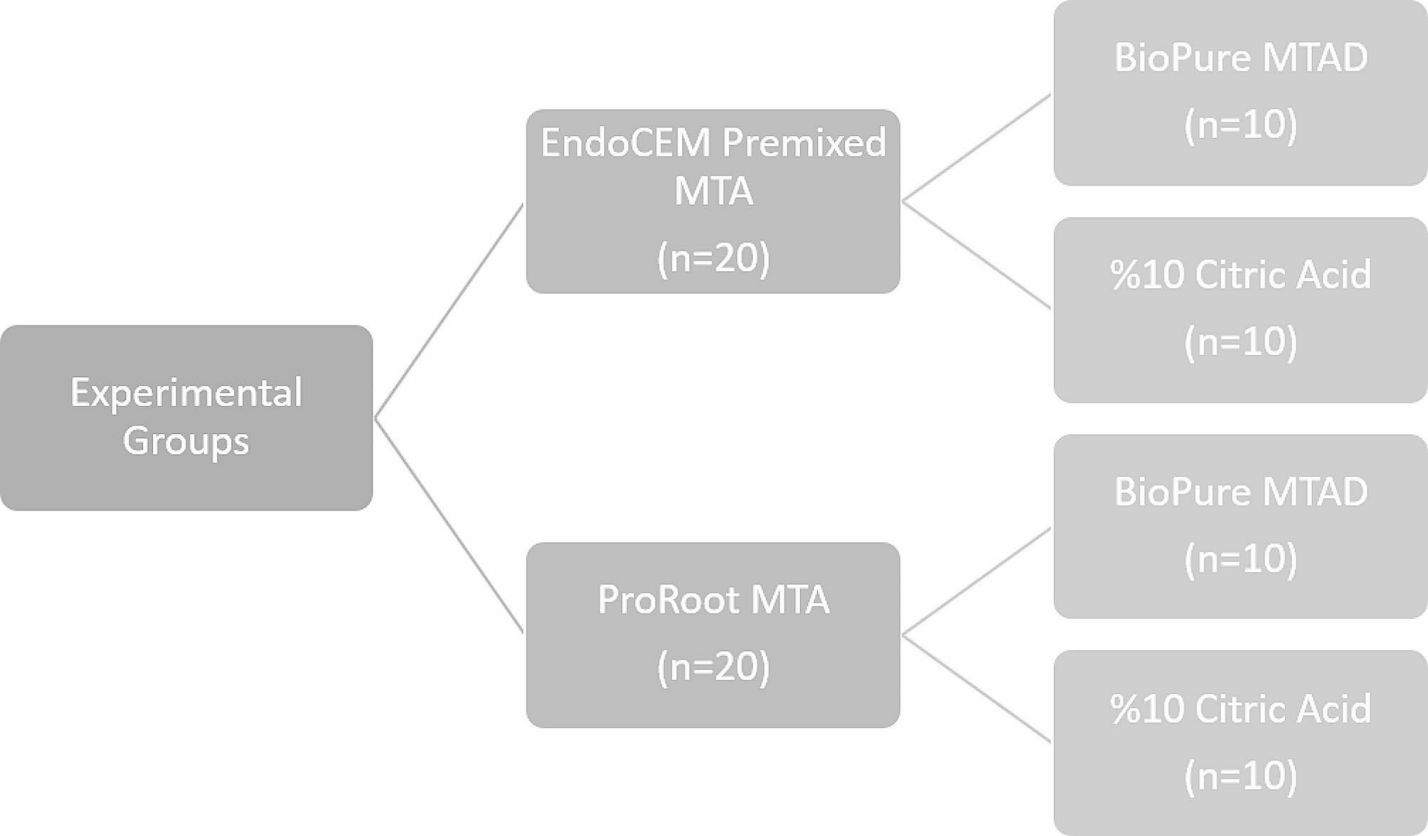
Experimental groups

Non-carious maxillary centrals (extraction indicated due to periodontal indications) were obtained from patients treated at Department of Oral Surgery of RTEU, who provided informed consent. Teeth with cracks, fractures, deformation, caries, internal/external root resorption and calcification were excluded from the study (Fig. 1).

The residues on the root surfaces were removed. Immature root simulation was performed by retrograde injection of No. 1–6 Peeso Reamers (Nic, Shenzhen, China). After the access cavity was prepared, calcium hydroxide was inserted as an intracanal medicament. A cotton pellet was placed on calcium hydroxide, and the teeth were temporarily restored with a glass ionomer. The samples were immersed in phosphate-buffered saline (PBS) and incubated at 37 °C for 2 weeks. The teeth were then randomly divided into 2 groups according to the presence of coronal barrier material (*n* = 20): ECM Premixed (Maruchi, Wonju, Korea) and PRMTA (Dentsply Tulsa Dental Specialties, Johnson City, TN, USA).

Calcium hydroxide was removed with 20 mL of 17% EDTA and 20 mL of saline. Surgicell was placed in the teeth, ending at 3–4 mm from the cementum-enamel junction. Then, the teeth were filled with the chosen barrier material up to the cemento-enamel junction. Periapical radiographs were taken for the control, and the material was restored with glass ionomer cement (Kavitan Pro, Spofa-Dental A.S., Czech Republic) and composite resin (Llis, FGM Produtos Odontológicas, Joinvile, SC, Brazil). The samples were immersed in PBS and kept in an incubator for two weeks before micro-CT analysis. Micro CT analysis is a non-destructive method that allows stepwise finely detailed 3D imaging of the same sample before and after removal, unlike destructive methods such as sectioning and SEM analysis. Using Micro CT scanning, the material in the root canal and dentin can be easily distinguished from each other, and the remaining material after retreatment can be easily visualized and measured quantitatively, providing superiority to qualitative measurement methods [15].

**Fig. 2:**
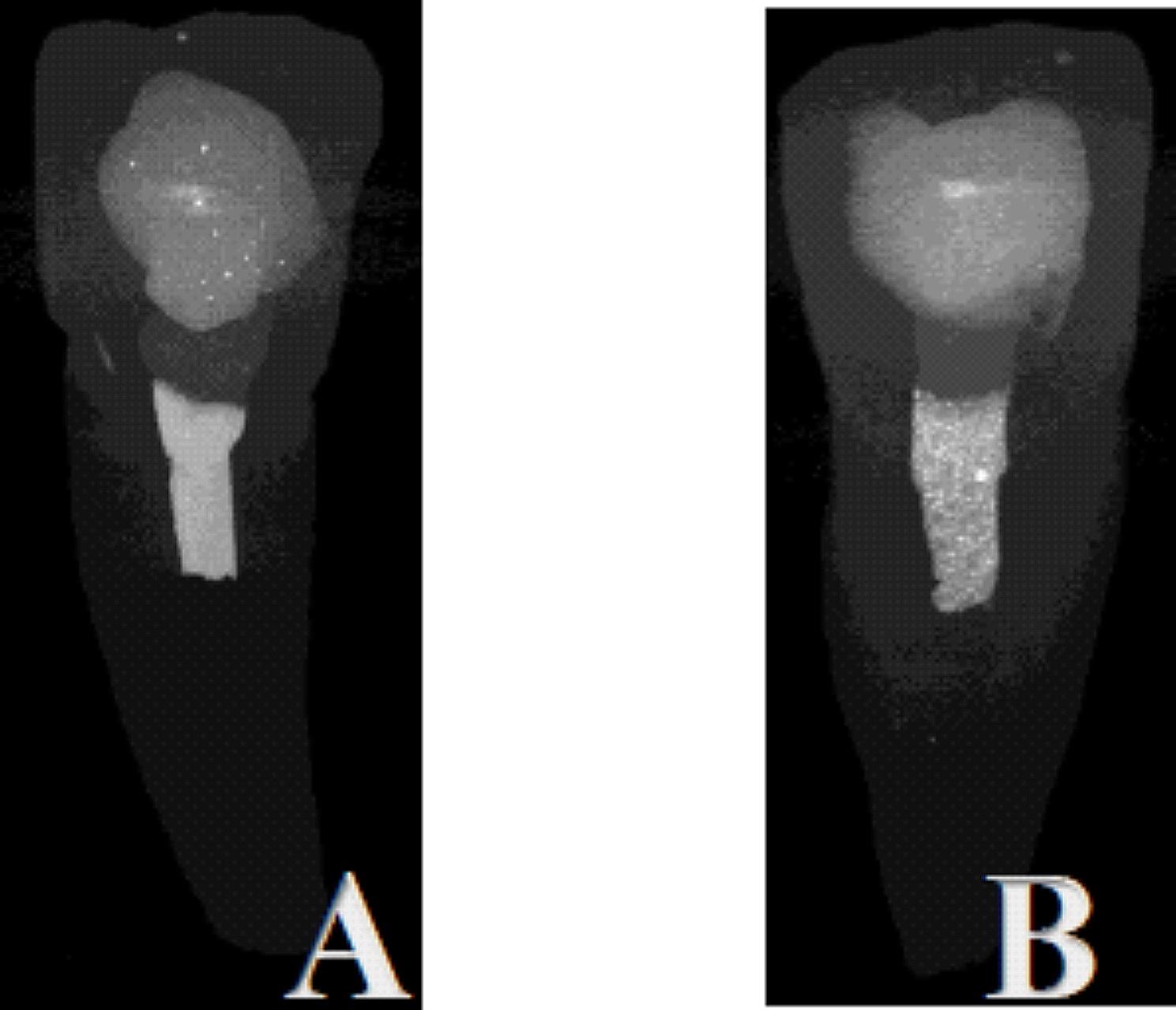
Micro-CT images: **(a)** ECM Premixed and **(b)** PR MTA

The samples were scanned with a SkyScan 1272 Micro-CT (Bruker Skyscan 1272, Billerica, Massachusetts, USA) at İnönü University Scientific and Technological Research Center. During the scans, the X-ray tube was operated at a voltage of 80 kV and a current of 124 µA. Scans were performed using a 180° rotation angle around the vertical axis, a 0.7° rotation range, an X-ray exposure time of 2400 ms, and a 1 mm Al + Cu filter. A total of 1752 sections with a mean thickness of 13.6 μm were taken from each root. The resulting images were repositioned in DataViewer (v.1.5.6.2; Bruker Corporation, Billerica, MA) software. Amd images were subsequently transferred to CTAn (v.18.4.0; Bruker Corporation, Billerica, MA) software for analysis. To calculate the initial volume (mm^3^) of the filling materials, the relevant areas were selected, and volumetric measurements were made and recorded (Fig. 2).

After micro-CT, the fillings in the samples were removed. Bust-05 and Bust-03 tips (mod: E, power setting: 10) were used in a Woodpecker DTE S6 ultrasonic device (DTE, Guilin Woodpecker Co., Guilin, Guangxi, China) for the removal of CSBMs in the coronal third. Ultrasonic instrumentation (UI) was applied under 1.5% NaOCl irrigation with 1–2 mm movements in the apicocoronal direction. UI for all teeth was performed blindly by a single operator under a dental operating microscope (Carl Zeiss, Germany). After it was determined by inspection that the material was removed, it was confirmed by radiography. After removal, the samples were scanned again via micro-CT with the same standards as those used for the first scan. The percentage ratio of the material volume obtained from this scan to the initial volume was recorded as the amount of remaining material after UI (%).

**Fig. 3:**
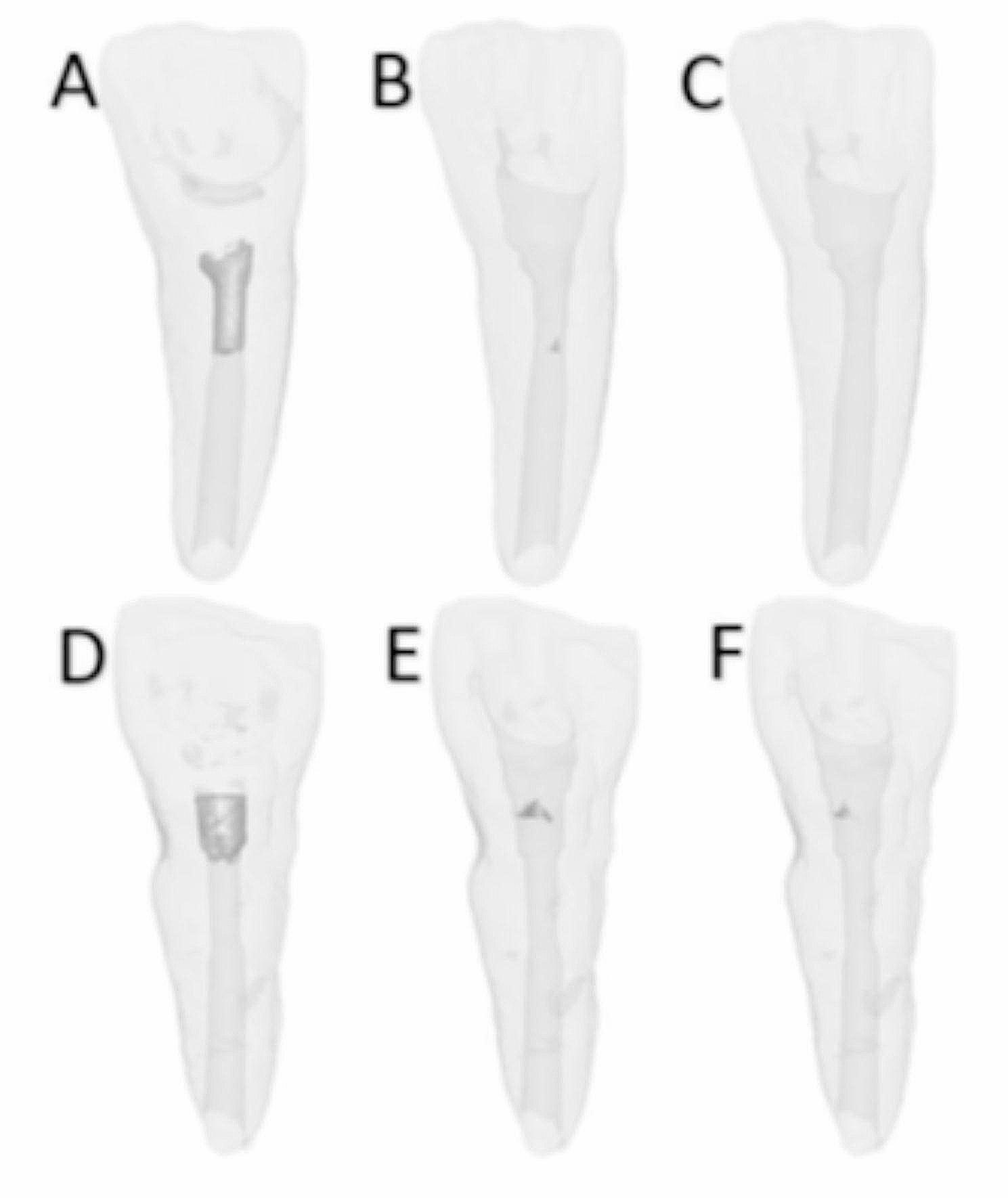
3D images of the initial and remaining materials: **(a)** ECM Premixed before removal, **(b)** after ultrasonic instrumentation, **(c)** after irrigation, **(d)** PR MTA before removal, **(e)** after ultrasonic instrumentation, and **(f)** after irrigation

**Fig. 4:**
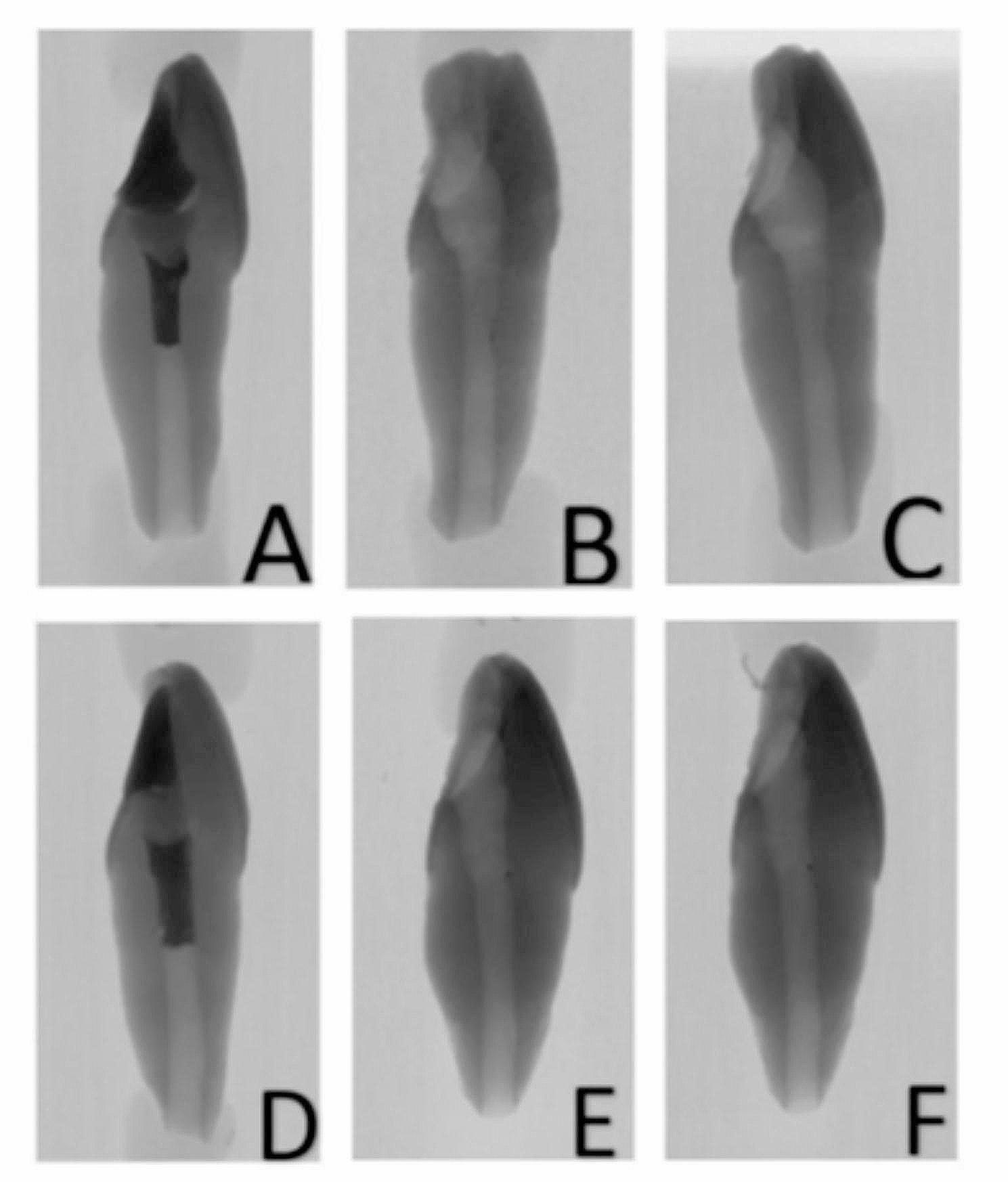
Micro-CT images: **(a)** ECM premixed before the procedure, **(b)** after ultrasonic instrumentation, **(c)** after irrigation, **(d)** PR MTA before the procedure, **(e)** after ultrasonic instrumentation, and **(f)** after irrigation

In the second stage of the study, to examine whether irrigation with acidic solutions had an additional effect on removal, the teeth in each material group were divided into 2 subgroups: MTAD and 10% CA solution (*n* = 10). Each canal was irrigated with 5 ml of solution for 5 min and then rinsed with 5 ml of saline. After the irrigation protocols, the samples were again scanned via micro-CT, after which the percentage ratio of the remaining material volume to the initial volume was recorded (%) (Figs. 3 and 4).

All the statistical analyses were performed in IBM SPSS 25. The assumption of normality of the coronal barrier material distribution was determined by the Shapiro‒Wilk test. While examining the initial volume values, an independent sample t test was applied because the data were normally distributed. The Mann‒Whitney U test was performed to compare the removal of materials after UI and to compare the removal of each material after UI with the removal after additional irrigation with acidic solutions. In addition, the Kruskal‒Wallis test was applied to compare the total removal of the material groups after irrigation with different aqueous solutions since there were more than three independent groups that did not follow a normal distribution. Post hoc corrected Bonferroni analysis was applied to identify the group or groups that caused the significant difference. The level of significance was set at *p* < 0.05.

## Results

The mean initial volumes of the CSBM were 5.98 mm³ for the ECM Premixed group and 6.59 mm³ for the PR MTA group. No statistically significant difference was detected between the two materials indicating similar amout of material to be removed for both groups (*p* > 0.05) (Fig. 2) (Table 1). The percentage ratio of the material volume measured after the removal procedure to the initial volume was used in the analysis of the removal efficiency. The amount of remaining material after UI was determined to be 0.7471% in the PR MTA group and 0.0093% in the ECM Premixed group (Table 2). As a result, significantly more ECMPremixed was removed compared to PRMTA (*p* < 0.05), while there was no significant difference between initial volumes.

**Table 1:** Comparison of initial volumes (mm³)

Material	Mean	Standard Deviation	Test Statistic	*p*
ECM Premixed	5.984	2.2888	− 0.867	0.392
PR MTA	6.595	2.1634		

**Table 2:** The amount of remaining material after ultrasonic instrumentation (%)

	Mean	Standart Deviation	Mean rank	Test Statistic	*p*
ECM Premixed	0.0093	0.01562	13.85	67.00**	0.000*
PR MTA	0.7471	1.55110	27.15		

When the material-solution groups were compared in terms of total removal capacity, significantly less remaining material was detected in the Premixed-MTAD group (0.0018%) than in the MTA-MTAD (0.1048%) and PR MTA-CA (0.0969%) groups (*p* = 0.024 and *p* = 0.004, respectively). Additionally, there was significantly less remaining material in the ECM Premixed-CA group than in the PR MTA-CA group (*p* = 0.013). No statistically significant difference was found between the other groups (*p* > 0.05) (Table 3). In each material group, there was no significant difference between the amount of remaining material after UI and after irrigation with MTAD or CA (*p* > 0.05) (Table 4).

**Table 3:** Comparison of remaining material after irrigation (%)

	Mean	Standard Deviation	Mean Rank	Test Statistic	*p*
**ECM Premixed-MTAD**	0.0019	0.00585	12.35	18.143***	0.000*
**ECM Premixed-CA**	0.0054	0.01336	14.10		
**PR MTA-MTAD**	0.1048	0.19141	26.40		
**PR MTA-CA**	0.0969	0.10396	29.15		

**Table 4:** Comparison of the remaining material (RM) after UI and after irrigation for each material (%)

	Solution		Mean	Standard Deviation	Mean rank	Test Statistic	*p*
**ECM Premixed**	MTAD	RM after UI	0.0074	0.0110	10.50	50.00	1.000
		RM after irrigation	0.0018	0.0058	10.50		
	CA	RM after UI	0.0111	0.0196	10.50	50.00	1.000
		RM after irrigation	0.0054	0.0133	10.50		
**PR MTA**	MTAD	RM after UI	0.9746	2.0229	10.50	50.00	1.000
		RM after irrigation	0.1048	0.1914	10.50		
	CA	RM after UI	0.5195	0.9936	10.50	50.00	1.000
		RM after irrigation	0.0969	0.1039	10.50		

## Discussion

The present study aimed to compare the removal efficiency of the ECM Premixed, which is used as a coronal barrier material in accordance with the RET procedure, and that of the PRMTA. CSBMs exhibit biomineralization by forming apatite-like crystalline precipitates inside the dentinal tubules, which leads to an interfacial layer at the material-dentin interface, enhancing the resistance to displacement of the material [16]. It was shown that this biomineralization process can be induced with PBS exposure in vitro [17]. Therefore, we kept the samples in PBS for two weeks before the removal procedure. Studies have shown that Peeso reamers, hand files, rotary files and retreatment files are insufficient for removing CSBM, while the use of ultrasonic instrumentation has been shown to be effective at removing these cements [10]. Thus, ultrasonic instrumentation was used for the removal of the materials in the present study. Methods such as sectioning, radiography and SEM analyses have been used for years in studies examining the efficiency of removal in the literature [17, 18]. These methods have lost popularity because they provide subjective data, and the samples may be damaged during these procedures, making reuse impossible [18]. Additionally, these methods do not allow precise measurement of the residual material volume [19]. Micro-CT is a noninvasive imaging method that has been used successfully in many fields of study in endodontics, such as for evaluating the cutting efficiency of files, evaluating retrograd filling materials and root canal obturation, and assessing the remaining material volume in the root canal [20–22]. Considering these advantages, in the present study, the remaining materials were examined via micro-CT. The amount of the remaining material was calculated as a percentage to minimize the effect of the initial material volume on the study findings. According to the micro-CT analysis, there was no significant difference between the initial volumes of the materials tested. However, the amount of remaining material in the ECM premixed group after UI (0.0093%) was significantly lower than that in the PRMTA group (0.7471%). Thus, the first null hypothesis of the study was rejected.

Since the studies comparing PRMTA and ECM Premixed were limited, we also considered the studies conducted with Endocem MTA (ECMTA), the powder-liquid form of ECM Premixed. However, in a study by Jang et al., it was reported that the ECM Premixed exhibited a more uniform and less rough surface, greater flowability, and lower film thickness than did the ECMTA Zr [23]. For this reason, it would not be correct to make a definitive comment about ECMPremixed based on the studies performed with ECMTA. However, this approach can be used to obtain a preliminary idea for further studies. In a study by Che et al., the compressive strength of PRMTA was significantly greater than that of ECMTA, and there was no difference in solubility between the two materials [24]. Kang et al. also reported that PRMTA has a greater compressive strength than does ECMTA [25]. Kim et al. examined the void volume formed when Micro-CT was used to introduce PRMTA, ECMTA, MTA Angelus, and Retro MTA as retrograde filling materials. The PRMTA group had the densest retrograde filling area with almost no pores in the material. In contrast, cross-sectional images of the ECMTA and Retro MTA groups showed decreased density and increased porosity. The results of that study suggested that ECMTA and RetroMTA might be less homogeneous than PRMTA is [26]. In light of these studies, the decreased removal of PRMTA may be associated with increased compressive strength, increased homogeneity, increased density and decreased porosity.

As a result of the bioactivity of CSBMs, apatite deposition occurs within the collagen fibrils leading formation of a mineral rich layer, and tag-like structures extending into the dentinal tubules, at the material-dentin interface [27]. Apatite nucleation and formation of tag-like structures decreases gaps at the interface and constitutes micromechanical bonding to dentin, improving the displacement resistance of the material [8, 28]. Reyes-Carmona et al. observed a high density of long tag-like structures at the interface and detected increased push-out bond strength when PRMTA was immersed in PBS, supporting the effect of the biomineralization process on resistance to dislodgement [16].

The apatite-like crystallins at the interface consist mainly of calcium and phosphorus [27, 29]. Han et al. stated that releasing higher amounts of Ca might increase the interfacial layer and tag-like structure formation of CSBMs, associated with the production of more calcium phosphate precipitate [30]. The calcium phosphate-forming capacity of CSBMs was found to be related to the chemical content, environmental conditions, and calcium-releasing properties [31]. The pozzolan-based CSBMs are associated with a decreased Ca release ability and Ca/P ratio of the apatite-like crystalline precipitates caused by pozzolanic reactions. In support of this, Adl et al. evaluated the dislodgement resistance of EndoSeal MTA, a pozzolan-based premixed material, in comparison with that of PRMTA and Biodentine and reported that EndoSeal MTA exhibited the significantly lowest bond strength values [32]. The authors attributed this result to the lower Ca release ability and Ca/P ratio of the apatite-like crystalline precipitates of pozzolan-based materials. Pozzolan is a siliceous material that does not show a significant cementitious value on its own. However, in finely divided form and in the presence of moisture, pozzolan cement increases the surface area and calcium silicate particles react with water to form cementitious compounds, such as calcium hydroxide and calcium silicate hydrate [33]. In the pozzolonic reaction, calcium hydroxide reacts with oxids of the pozzolan, and forms additional calcium silicate hydrate and calcium aluminate hydrate. In this process the free calcium hydroxide is consumed and gradually reduces in amount, which may lead to a decrease in the Ca/P ratio of the precipitates [34, 35]. In a study by Han et al., after immersion in PBS for two weeks, WMTA released significantly more Ca and produced apatite-like crystalline precipitates with higher Ca/P ratios than did puzzolan-based ECMTA and ENDOCEM Zr, indicating greater bioactivity [35]. In line with these studies, the greater removal of ECM PRremixed in the present study might be attributed to pozzolanic reactions.

The effects of different solvent solutions on the microhardness of CSBMs have been investigated in many studies. It was shown that 2% carbonic acid, 10% CA and 20% tartaric acid caused a significant decrease in the MTA microhardness [10, 27]. In a study examining the effect of chelating agents on the surface properties and solubility of MTA, MTAD removed more material, increased the surface roughness, and extracted more calcium than EDTA did [11]. In the second stage of the present study, MTAD and 10% CA were used to examine whether they had significant effects on material removal. Considering the results of previous studies, the solutions were applied for 5 minutes to determine the effect of the solutions on the CSBM and to preserve the microhardness of the dentin [10, 11, 28]. As a result, MTAD and 10% CA did not significantly increase the removal of PRMTA or ECMPremixed. Therefore, the second null hypothesis of the study was accepted. This result may be related to the duration of exposure to acidic solutions. Butt et al. reported significantly reduced microhardness of WMTA after 10 and 20 min exposure to 10% CA, at 1. and 21. day of setting [11]. Smith et al. reported that 5 min irrigation with BioPure MTAD resulted in only minor volume reductions of the set PR MTA, and a significant correlation between dissolution of MTA and the exposure time. The authors also predicted that 32 h’ continuous irrigation would be required for BioPure MTAD to dissolve a 2-mm-thick layer of PR MTA [12]. In line with these studies, the absence of significant effect of the CA and MTAD on the removal of CSBMs may be attributed to the relatively short exposure time of 5 min, preferred to prevent destructive effects of the solutions on the dentin.

Considering the statistical aspect of the study, in the comparison of material removal after ultrasonic instrumentation (UI) and the subsequent removal of each material following additional irrigation with acidic solutions, it was observed that the distribution was not normal within the groups. Similarly, when comparing the total removal of material groups after irrigation with different aqueous solutions, the distribution did not adhere to a normal distribution. Consequently, non-parametric tests were employed for these analyses. While a low standard error is anticipated due to the high standardization ability of the in-vitro studies, it may not be feasible to apply irrigation and material removal similarly for each sample. This limitation is associated with the standardization aspect of the study.

This study may not fully reflect the clinical conditions due to the limitation of its in vitro design. The bioactivity of CSBMs is closely correlated with their chemical reaction with body fluids in a manner compatible with the repair processes of the tissue. The biological fluids constantly provides new phosphate and may increase the amount of apatite formed on the cement surface [36].The ion composition of the environment affects the composition of precipitates at the material dentin interface. The closer the ion concentration is to that in biological body fluids, the formed apatite layer will have the similar chemical and structural properties to those formed in the physiological process in vivo [37]. Additionally, the difference in ion concentration between PBS and physiological tissue fluid may cause a normally bioactive material to fail to form apatite in vitro, or vice versa. Therefore, although many studies have suggested the use of PBS as a widely accepted method to stimulate the bioactivity of CSBMs, clinical inferences derived from the results with simulated body fluids should be interpreted with caution. In future studies, more attention should be paid to examining to what extent the interaction between the material and the oral environment affects the bioactivity of CSBMs.

Another limitation of this study may be that no examination was conducted in terms of damage to the dentin surface during removal procedures. Ultrasonic application may lead to cracks on the dentin surface depending on the type and power of the ultrasonic tip, and duration of application [38].Besides, as aforementioned, exposure to acidic solutions such as citric acid and MTAD, may lead dentin damage depending on the application time [38].In retreatment cases, preserving the remaining tooth tissue is important for the prognosis of the tooth, as well as the complete removal of materials, especially in immature teeth with thin dentin walls. Therefore, further studies examining the root dentin for cracks, or deformation after material removal may guide clinicians in choosing the safe and effective method for the removal of CSBMs.

## Conclusions

Within the limitations of this study, a great deal of the material was removed by the UI under an operating microscope. ECM Premixed was removed more effectively than was PRMTA. Moreover, acidic solutions did not provide any significant benefit in terms of material removal for either CSBM.

## Data Availability

All data generated or analysed during this study are included in this published article [and its supplementary information files].
